# Secondary cytoreductive surgery in recurrent clear cell carcinoma of the endometrium: A case report

**DOI:** 10.1016/j.ijscr.2023.108412

**Published:** 2023-06-16

**Authors:** Connie Cheng, Nicole Jenkins, Noelle Aikman, Karim ElSahwi

**Affiliations:** a Department of Obstetrics and Gynecology, Jersey Shore University Medical Center, 1945 Route 33, Neptune, NJ 07753, United States of America; b Division of Gynecologic Oncology, Department of Obstetrics and Gynecology, Jersey Shore University Medical Center, 1945 Route 33, Neptune, NJ 07753, United States of America

**Keywords:** Case report, Secondary cytoreduction, Secondary cytoreductive surgery, Secondary debulking, Endometrial cancer, Clear cell carcinoma, Minimally invasive surgery

## Abstract

**Introduction & importance:**

Endometrial cancer with high-risk histology is associated with a majority of recurrences and death. However, unlike other cancers, such as ovarian, there is a paucity of research demonstrating the benefits of secondary cytoreduction. In this case report we aim to aid in identifying individuals who may be ideal candidates for secondary cytoreduction surgery after minimally invasive hysterectomy and staging by a gynecologic oncologist at an academic institution and diagnosed with clear cell endometrial cancer.

**Case presentation:**

A 72 year-old female patient presented with postmenopausal bleeding and was subsequently diagnosed with Stage IIIC2 clear cell carcinoma of the endometrium. She represented 20 months after receiving initial staging and adjuvant chemotherapy with increasing CA-125 levels and radiographic evidence of left para-aortic lymph node oligo metastasis. She underwent secondary cytoreductive surgery via robotic-assisted laparoscopic para-aortic lymph node dissection and salvage chemotherapy. After 45 months of follow-up physical exam, CA-125 levels and CT of the abdomen and pelvis have remained without evidence of disease.

**Clinical discussion:**

We review the literature on secondary cytoreductive surgery (SCS) in endometrial cancer (EC) to identify factors associated with improved survival.

**Conclusion:**

Secondary cytoreduction in endometrial cancer may lead to prolonged progression-free survival in well-selected patients.

## Highlights

• Secondary cytoreductive surgery (SCS) in well selected patients with recurrent endometrial cancer can confer overall survival benefit.
• Factors associated with improved survival after SCS have been retrospectively identified and should be taken into consideration.
• Prospective validation of factors associated with improved survival after SCS in EC is needed.

## 1 Background

Endometrial cancer (EC) is the most common gynecological cancer in the United States with an estimated incidence of 65,950 new cases and 12,550 deaths in 2022 [1]. It is classified into Type I or low-grade endometrioid cancer, and Type II which comprises grade-3, clear cell, uterine serous cancer, and carcinosarcoma. Among the two types of endometrial carcinoma, Type II occurs less frequently and is associated with poorer outcomes due to its propensity for extrauterine spread and recurrence [[2], [3], [4]].

The 5-year overall survival (OS) rate of patients with clear cell endometrial cancer and a higher International Federation of Gynecology and Obstetrics (FIGO) stage is <50 % [5]. Management options for any type of recurrent endometrial cancer (EC) traditionally included surgical resection, cytotoxic chemotherapy, radiation therapy, hormonal therapy, or a combination, multi-modal approach. Historically, surgical management involved exenterative procedures for central pelvic recurrences. There is evidence that non-exenterative secondary cytoreductive surgery (SCS) may be associated with improved survival compared to medical management in recurrent regional and distal disease. The range OS of non-exenterative SCS based on compilation of studies is 13–35 months and the range of progression-free interval (PFI) of 13–20 months [7]. We present a case that demonstrates a prolonged survival benefit after SCS in nodal recurrent advanced-stage clear cell endometrial cancer. This work has been reported in line with the SCARE criteria. The patient's consent has been obtained. According to the local Internal Review Board rules, this work has been exempt from a full review.

## 2 Case presentation

**Fig. 1:**
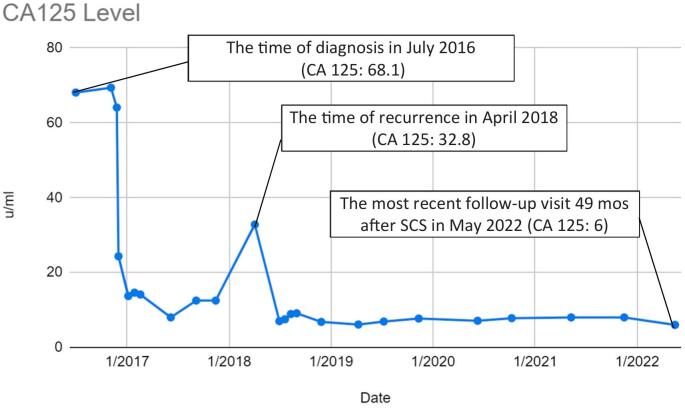
Trend in CA-125 levels from initial diagnoses, primary debulking/chemotherapy, secondary debulking/chemotherapy and current disease remission.

A 72-year-old, para one Caucasian female, presented with postmenopausal bleeding in July 2016. Her past medical history was significant for hypertension managed by Hydrochlorothiazide. She had no family history of ovarian, uterine, breast, pancreas, colon, or prostate cancer. Social history included social drinking with 1–2 drinks per week, with no smoking or recreational substance use. Her BMI was 27, and Eastern Cooperative Oncology Group Performance Status (ECOG PS) score was 0, denoting that she was fully active and able to perform activities without restriction. The ECOG PS is a scale describing a patient's functional status regarding their ability to take care of themselves and perform daily and physical activities. An endometrial biopsy showed clear cell carcinoma of the endometrium. A CT-Abdomen/Pelvis (CT-AP) was consistent with thick endometrium, unremarkable adnexal structures, and no evidence of metastatic disease. CA19-9 and CA-125 were 168.9 μ/mL and 68.1 μ/mL respectively. Upon final review of the patient's history and physical, she was found to be optimized for surgery. She underwent a robot-assisted total laparoscopic hysterectomy, bilateral salpingo-oophorectomy, omentectomy and pelvic and para-aortic lymph node dissection by a gynecologic oncologist. Pathology showed mixed endometrioid and clear cell adenocarcinoma with 90 % myometrial invasion, positive metastatic adenocarcinoma to multiple pelvic and paraaortic lymph nodes bilaterally, no omental metastasis, and negative pelvic washings. This was consistent with FIGO stage IIIC2, grade 2 endometrioid adenocarcinoma/clear cell carcinoma. She was treated with adjuvant chemotherapy with platinum and taxane doublet IV every 21 days for a total of six cycles. During immediate post-chemotherapy surveillance, there was no evidence of residual disease on CT scan, and serial CA 125 levels decreased appropriately. Twenty months after initial debulking, CA125 levels were noted to double over a five-month period (Fig. 1). Patient had no presenting symptoms and the physical exam was without evidence of recurrence.

## 3 Investigations

**Fig. 2:**
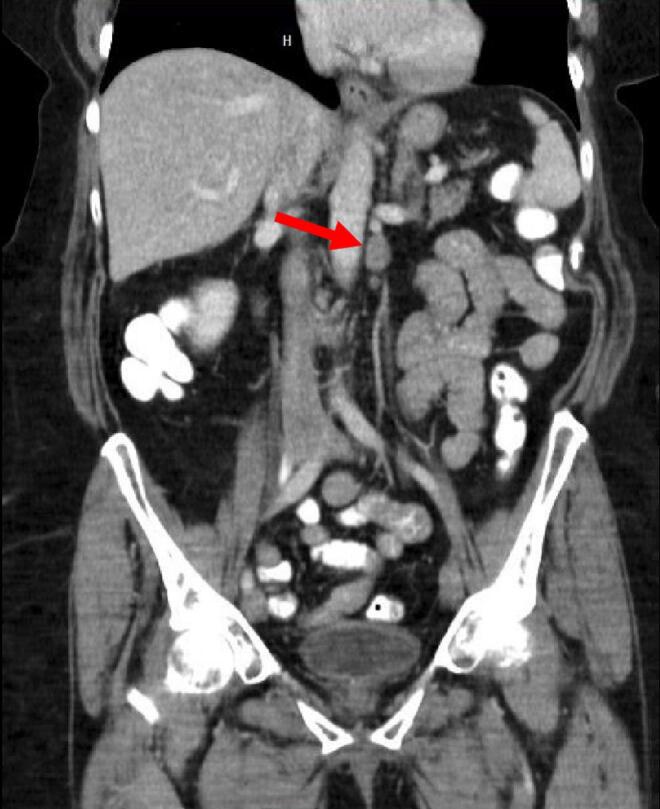
Left para aortic lymph node enlargement measuring 12 × 17 mm (arrow).

A CT-AP was performed, demonstrating interval growth of a left para-aortic lymph node measuring 12 × 17 mm (Fig. 2). Recurrence of disease from previously resected endometrial carcinoma was suspected.

## 4 Treatment

Options for management including risks, benefits, and potential complications were discussed. She opted for a secondary cytoreductive surgery and underwent a robot-assisted left para-aortic lymph node dissection. Pathology showed metastatic undifferentiated endometrioid adenocarcinoma in one of seven lymph nodes. Immunohistochemistry showed tumor cells positive for Estrogen and Progesterone receptors (ER and PR) with patchy Human Epidermal Growth Factor (HER2) expression as well as deficient DNA mismatch repair protein expression; negative for MLH1 and PMS2 expression. Methylation was positive, suggestive of sporadic somatic gene alteration. Pelvic washings were negative for malignant disease. Salvage chemotherapy was initiated with platinum and taxane doublet every 21 days for an additional four cycles.

## 5 Outcome and follow-up

CT scan immediately following salvage chemotherapy showed no evidence of disease and CA125 decreased to 8 μ/ml. Currently, surveillance with periodic physical exams and serial tumor markers has demonstrated no evidence of recurrent disease. The progression-free interval (PFI) from the date of the SCS to the date of her last visit is 48 months. Her overall survival (OS) is 68 months. She remains on scheduled biannual follow-up.

## 6 Discussion

We present the case of a 72-year-old patient with good performance status which developed a solitary nodal recurrence 20 months after uncomplicated minimally invasive staging and chemotherapy for stage IIIC2 clear cell carcinoma of the endometrium. She was successfully treated with minimally invasive SCS and salvage chemotherapy. She remains without evidence of disease 48 months after salvage treatment.

Retrospective studies and meta-analyses (Table 1) have identified SCS as a predictor of improved survival in recurrent EC [[9], [10], [11]]. The OS for patients who underwent SCS in one study [4] was 56 months, consistent with the 68-month OS we are reporting in our patient. Importantly, the OS was significantly improved when complete cytoreduction was achieved. For example, Barlin et al. calculated that every 10 % increase in cytoreduction improved survival by 9.3 % (p = 0.04) [9]. Factors associated with achieving optimal SCS in another study included solitary tumors, small size (<6 cm), and younger patients (<56 years old) [7]. Younger age at diagnosis has been consistently associated with better survival outcomes after SCS for EC with a cut off 70 years of age [12]. Shikama et al. [10] identified ECOG PS as a predictor of improved survival in SCS in EC and a randomized controlled trial (DESKTOP III) in ovarian cancer has validated ECOG PS as a deciding factor when considering SCS [13]. Our patient had a small solitary para-aortic lymph node, although she was 72 years-old. However, we believe age to be a surrogate for performance status which likely contributed to the improved survival demonstrated by our patient.

**Table 1:** Review of the literature summarizing factors associated with improved survival after SCS for recurrent EC.

Year	Article	Factors statistically significant for improved survival with SCS	Overall survival (SCS vs none)
1998	Scarabelli et al. [14]	- Resection to no macroscopic disease	11.8 mons vs undefined
2003	Campagnutta et al. [15]	- Less residual disease - Use of postoperative chemotherapy - Central pelvic recurrence	53 mons vs 9 mons
2006	Awtrey et al. [16]	- Size of residual disease	43 mons vs 10 mons
2006	Bristow et al. [17]	- No grossly visible disease	39 mons vs 13.5 mons
2010	Barlin et al. [11]	- Complete cytoreduction to no gross residual disease	Each 10 % increase in SCS improves survival time
2014	Ren et al. [7]	- <56 years of age - Tumor size <6 cm - Solitary recurrence	–
2019	Shikama et al. [10]	At primary cytoreductive surgery: - FIGO I/II - No pelvic and/or paraaortic lymph node metastasis - Endometrioid histology - Before secondary cytoreductive surgery: - Longer tumor free interval (≥24 mons) - ECOG 0 At secondary cytoreductive surgery: - No residual tumor	21 mons vs 14.8 mons
2021	Moukarzel et al. [6]	- <70 years of age at initial diagnosis - Progression-free survival >19 months - FIGO Grade 1 or 2 - Endometrioid or clear cell histology - Early stage 1 or 2 disease - No residual disease at initial surgery - Short length of stay from 0 to 6 days - No more than two grade 3 complications	57.6 mons vs 24.5 mons

Other patient factors associated with improved survival after SCS include endometrioid/clear cell histology, a longer progression-free interval (>19 months), low grade, and early stage [12]. The absence of residual disease at initial surgery was identified by Mourkazel et al. [6] as a predictor of a successful SCS. Indeed, our patient had Grade 2/clear cell cancer and a progression-free interval of 22 months. Although she had stage IIIC2 disease, this was due to metastasis localized to lymph nodes that were removed on initial surgical staging. Table 2 offers a comparison of our patient to the criteria proposed by Mourkazel et al.

**Table 2:** Comparison between presented case and patient criteria suggested by Moukarzel et al for considering SCS in recurrent EC [6].

Factors	Moukarzel et al. [6]	Presented case
Age	<70	72
Progression free survival	>19 months	22 months
FIGO	Grade 1 or 2	Grade 2
Histology	Endometrioid or clear cell histology	Endometrioid adenocarcinoma/clear cell carcinoma
Stage	Early stage 1 or 2 disease	Stage IIIC2
Residual disease at initial surgery	None	None
Length of stay	0 to 6 days	<1 day
Complications	≤2 Grade 3	None

Finally, surgical technique and approach deserve special mention. An uncomplicated initial surgical resection followed by a short hospital stay has been associated with improved outcome after SCS [10]. Furthermore, SCS was associated with an acceptable complication rate in most studies [7,[9], [10], [11]]. Although the surgical approach was not specified in the literature, this is the first reported case, to our knowledge, that was managed with minimally invasive surgery (MIS) in both the initial and recurrent settings. We believe that this approach when feasible, along with ERAS protocols would contribute to the favorable outcomes observed. The patient presented is relatively healthy with ECOG 0, thus limiting application of SCS in recurrent endometrial cancer to similarly well optimized patients.

We conclude that non-exenterative SCS in EC merits further prospective evaluation. MIS has obvious advantages that may contribute to more favorable patient outcomes; therefore, future studies should consider including this option when feasible.

## 7 Learning points/take home message

• Secondary cytoreductive surgery (SCS) in well-selected patients with recurrent endometrial cancer can confer overall survival benefits.
• Factors associated with improved survival after SCS have been retrospectively identified and should be taken into consideration.
• Prospective validation of factors associated with improved survival after SCS in EC is needed.

## Ethical approval

Ethical approval for this study was provided by the Internal Review Board of Hackensack Meridian Health Jersey Shore University Medical Center, Neptune, New Jersey in the United States of America on April 3, 2022.

## Funding

There was no funding for this research.

## CRediT authorship contribution statement

Dr. CC, Dr. NJ, Dr. NA, and Dr. KE all provided integral contributions to make this case report possible. Dr. KE was the primary surgeon who cared for this patient. The idea for this article was formulated equally by all three authors. The literature search was carried out by Dr. CC and Dr. NJ. The article was drafted by Dr. NJ and Dr. CC, with editorial support provided by Dr. KE and Dr. NA.

## Guarantor

Karim ElSahwi

## Consent

Written informed consent was obtained from the patient for publication of this case report and accompanying images. A copy of the written consent is available for review by the Editor-in-Chief of this journal on request.

## Conflict of interest statement

None of the authors have conflict of interest.
